# Using sutures to attach miniature tracking tags to small bats for multimonth movement and behavioral studies

**DOI:** 10.1002/ece3.1584

**Published:** 2015-07-04

**Authors:** Kevin T Castle, Theodore J Weller, Paul M Cryan, Cris D Hein, Michael R Schirmacher

**Affiliations:** 1 Wildlife Veterinary ConsultingFort Collins, Colorado; 2 Pacific Southwest Research Station, United States Department of Agriculture Forest ServiceArcata, California; 3 Fort Collins Science Center, United States Geological SurveyFort Collins, Colorado; 4 Bat Conservation InternationalAustin, Texas

**Keywords:** Data logger, *Eptesicus fuscus*, geolocator, GPS tracking, *Lasiurus cinereus*, migration, movement ecology, satellite tracking, telemetry

## Abstract

Determining the detailed movements of individual animals often requires them to carry tracking devices, but tracking broad-scale movement of small bats (<30 g) has been limited by transmitter technology and long-term attachment methods. This limitation inhibits our understanding of bat dispersal and migration, particularly in the context of emerging conservation issues such as fatalities at wind turbines and diseases. We tested a novel method of attaching lightweight global positioning system (GPS) tags and geolocating data loggers to small bats. We used monofilament, synthetic, absorbable sutures to secure GPS tags and data loggers to the skin of anesthetized big brown bats (*Eptesicus fuscus*) in Colorado and hoary bats (*Lasiurus cinereus*) in California. GPS tags and data loggers were sutured to 17 bats in this study. Three tagged bats were recaptured 7 months after initial deployment, with tags still attached; none of these bats showed ill effects from the tag. No severe injuries were apparent upon recapture of 6 additional bats that carried tags up to 26 days after attachment; however, one of the bats exhibited skin chafing. Use of absorbable sutures to affix small tracking devices seems to be a safe, effective method for studying movements of bats over multiple months, although additional testing is warranted. This new attachment method has the potential to quickly advance our understanding of small bats, particularly as more sophisticated miniature tracking devices (e.g., satellite tags) become available.

## Introduction

**Figure 1:**
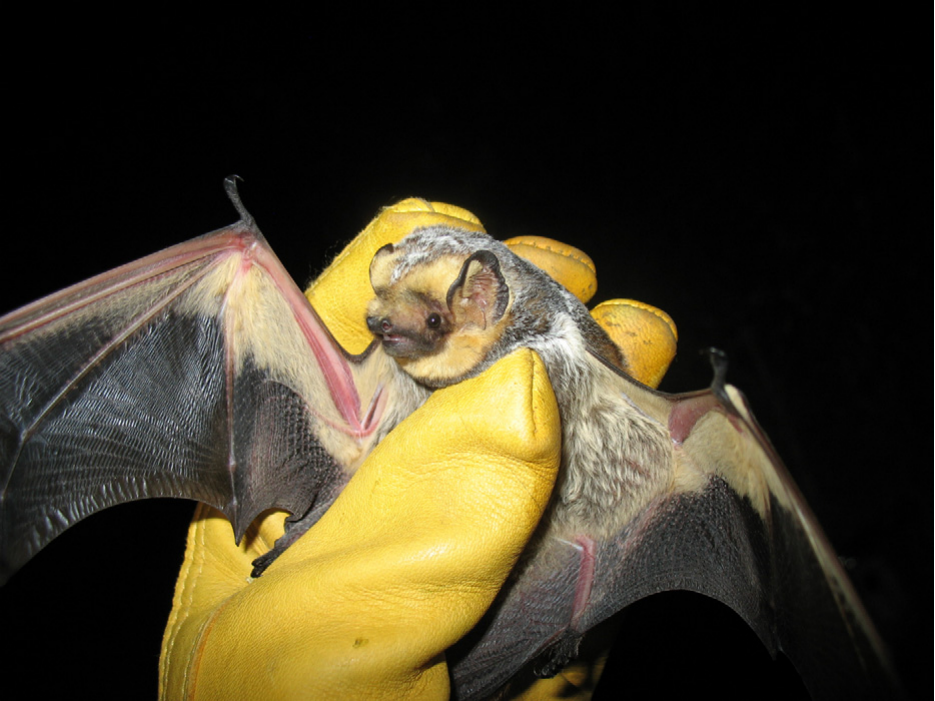
Hoary bat (*Lasiurus cinereus*), one of the species of bat to which miniature tracking tags were attached in this study. Photo credit P. Cryan.

Bats are important components of many ecosystems, with ecological roles including insectivory, pollination, and dispersal of nutrients between ecosystems (Kunz et al. 2011). Their evolutionary success is attributable to their ability to fly in the dark, but this characteristic makes it extremely difficult to study their behavior. Because bats conceal themselves during the day and are difficult to observe at night, we know little about where even common and widespread species, most of which are small (<30 g), spend certain times of year. Further, the habits of most temperate-zone bat species outside the summer season are poorly documented (Weller et al. 2009). Hoary bats (*Lasiurus cinereus*) range across much of North America during the summer months, but details of their continental migrations or location of their wintering grounds are limited (Cryan 2003; Baerwald et al. 2014; Cryan et al. 2014) (Figure 1). Lack of information about migratory movements and seasonal habitat use hinders efforts to understand impacts of human activities, disease, or other stressors on bat populations (Fleming and Eby 2003; Messenger et al. 2003; Kunz and Racey 2009). For example, hoary bats comprise approximately 38% of the hundreds of thousands of bat fatalities occurring at wind turbines in the United States and Canada each year, and most of these fatalities occur during their autumn migration and mating period (Arnett and Baerwald 2013). Knowledge of the behavior of individual bats during migration may help to elucidate why they are susceptible to death at wind turbines and ultimately could help to mitigate this concern. In the context of disease ecology and public health, being able to precisely follow the movements of individual bats could help advance understanding of pathogen spread for diseases that affect bats and also sometimes humans (e.g., white-nose syndrome, rabies).

Precise movements of bat species are mostly unknown because biologists have lacked the technology and methods of tag attachment to monitor bats over long time periods or distances (>20 km: Cryan and Diehl 2009; Holland and Wikelski 2009). In addition, the potential negative impacts of tags on bats are poorly understood (Aldridge and Brigham 1988; O’Mara et al. 2014). Little is known about the effects of size, weight, tag configuration, and method of attachment on behavior and maneuverability. Trade-offs among the duration a device is attached, the welfare of the animal, and quantity and quality of information obtained are important considerations.

The small size of most bats limits the types of devices that can be used to follow their movements (Cryan and Diehl 2009; Holland and Wikelski 2009; Popa-Lisseanu and Voight 2009). GPS and satellite devices have been used successfully on larger bats of the family Pteropodidae (Richter and Cumming 2008; Smith et al. 2011; Tsoar et al. 2011), but the tracking of smaller bats has been limited to small (<1 g) very high frequency (VHF) radio transmitters. Because the reception distance of these VHF transmitters is short, the ability to track long-distance movements of bats is limited. To our knowledge, few insectivorous bats have been successfully tracked using VHF radio transmitters more than about 500 km (Britzke et al. 2006; Holland et al. 2006; Dechmann et al. 2014), and such efforts can be expensive because they typically involve the use of aircraft to find and follow bats.

Recent advances in technology have resulted in production of small devices (<1.5 g) capable of recording information from small animals over extended time periods. For example, “light-level” geolocators have been used to record migratory movements of birds (Bächler et al. 2010). Recently, GPS tags capable of logging animal positions over the course of a full-year and miniature data loggers that allow documentation of activity periods and flight heights have been developed (Liechti et al. 2013). Currently, these devices directly or indirectly record location data, but the data cannot be remotely accessed in real-time, so devices must be recovered to retrieve information. The size, reliability, and functionality of such devices are likely to improve in the future and enhancements will likely allow real-time, remote data collection. Although these new opportunities for studying long-term movements of small bats are promising, they are accompanied by the challenge of attaching the tags to bats for long periods of time. Successful use of tracking devices requires that they be attached in a manner that minimizes impacts to the animal’s health and behavior, but few alternatives for such attachment have been developed.

The most common method of attaching tracking devices to small bats involves using surgical glue to attach a transmitter to the dorsum, between the scapulae (Amelon et al. 2009). This method is widely used, with few reported injuries or deaths attributable to tags (Amelon et al. 2009; O’Mara et al. 2014); however, few studies have assessed the health effects of tag attachment (Kurta and Murray 2002; Neubaum et al. 2005; Patriquin et al. 2010; O’Mara et al. 2014). Glued transmitters rarely remain on a bat’s back for more than 3–4 weeks and often fall or are groomed off after much shorter periods (e.g., mean duration = 9.3 ± 4.6 days, O’Mara et al. 2014). This eventual shedding of the tracking device is a desirable outcome because it minimizes long-term impacts to the bat.

Longer term attachment of tracking devices has been achieved using harnesses on birds and collars on mammals, including bats. Harnesses are not feasible for use on bats because they require puncturing the delicate wing membrane bats use for flight and prey capture. Collars, sometimes combined with glue, have been used to substantially increase the duration of tag attachment (mean duration = 163.1 ± 13.2 days, O’Mara et al. 2014), but the attachment durations were still too short to be effective for monitoring yearly movements of bats. Collars also can be removed by the bat or conspecifics, can become entangled with the bat’s head or legs or objects in the environment, may rotate and negatively affect the bat’s movements, and affect echolocation (Supplemental Information).

Our objective was to test the feasibility of suturing GPS tags and data loggers to small bats (<30 g) as a means of tracking their long-distance movements over time. Our ultimate goal was year-long deployment (and subsequent retrieval) of tracking tags on small bats that migrate long distances (>1000 km), such as hoary bats. Because we lacked well-validated methods for attaching such devices to bats for extended periods of time, we first experimented with suturing tags to bats over shorter time periods (weeks–months) and evaluated their effects on bat health.

## Methods

Capture and sampling of bats followed guidelines of the American Society of Mammalogists (Sikes et al. 2011), and appropriate state collection permits were obtained. Capture and suturing protocols were approved by the Institutional Animal Care and Use Committee of the U.S. Geological Survey, Fort Collins Science Center (Protocol 2014-08 and Standard Operating Procedure 01-01 for Capture, Handling, Marking, Tagging, Biopsy Sampling, and Collection of Bats).

**Figure 2:**
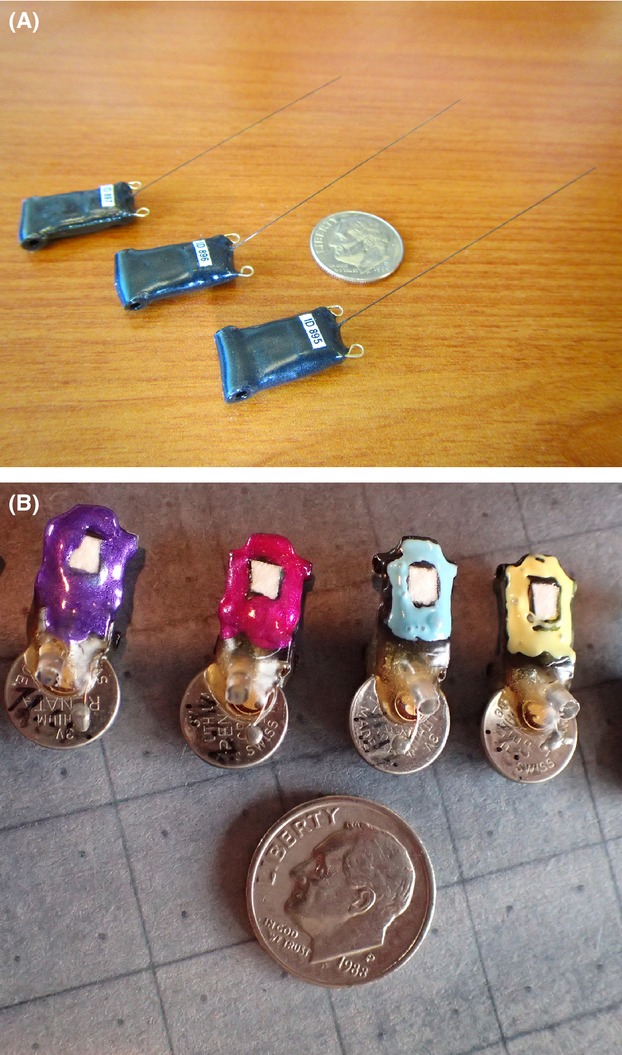
(A) Global positioning system (GPS) tracking tags that were experimentally sutured to big brown bats (*Eptesicus fuscus*) and hoary bats (*Lasiurus cinereus*) to track their long-term movements. Coin diameter = 18 mm. (B) Data logger tags sutured to hoary bats to track their long-distance movements. Please see text for technical specifications of GPS and data logger tags. Coin diameter = 18 mm.

We developed a suturing method to attach three types of tracking tags to wild bats: programmable GPS tags with and without VHF transmitters (Pinpoint 8, Lotek Wireless, Newmarket, ON, Canada) and data logger tags (F. Liechti, Swiss Ornithological Institute, Sempach, Switzerland) (Fig.2). The GPS-only tags weighed 1.1 g and had dimensions of 22.0 mm × 11.0 mm× 4.5 mm with a posterior-extending antenna 43 mm in length. The GPS tags were programmed to record their location on 8 specified dates and times. Five GPS tags also included VHF transmitters (PicoPip AG317; Lotek Wireless, Newmarket, ON, Canada). Those tags weighed 1.4 g and had dimensions of 20.5 mm × 15.0 mm × 6.0 mm with an additional posterior-extending antenna 145 mm in length. VHF transmitters were programmed to produce 30 pulses per minute over a 38-day period. The range of VHF transmitters was approximately 300 m and was primarily intended to help establish which animals were still in an area following tag attachment. Both types of GPS tags were rechargeable and reprogrammable without removing the tag from the bat. We programmed GPS tags to record nighttime locations approximately 1 hour after local sunset and daytime locations at noon. We also attached multifunction data loggers to hoary bats. These tags weigh 1.14 g and had dimensions of 24.0 mm × 10.0 mm × 4.0 mm with a dorsocaudal extending light sensor measuring 5 mm. Data loggers recorded light levels, air pressure, acceleration, and temperature. We attached data logger tags to evaluate our device attachment methods and to assess future applications of the technology. Data loggers required removal to obtain stored data.

We used a monofilament synthetic absorbable suture (PDS II, polydioxanone; Ethicon, Inc., Cincinnati, OH), which has minimal reactivity in tissue and is specified to dissolve by hydrolysis after 182–238 days (Ethicon product insert). This expected suture-failure duration falls short of our goal of year-long tag attachment, but errs on the side of caution in considering the bat’s health during this early testing phase. Longer lasting suture materials exist (e.g., nonabsorbable synthetic polymers or stainless steel), but we wanted to assure that if the bats were not recaptured, the tags would eventually fall off and not cause any permanent impediment.

We practiced suture methods and optimized placement on a bat’s body in the laboratory by suturing 1.0 g epoxy and wire mock-ups of GPS tags to bat carcasses obtained from public health departments and wind energy facilities. To field test the safety and efficacy of suturing tags to live bats, we captured big brown bats from known roost locations with high recapture rates in Fort Collins, Colorado (Ellison et al. 2007; O’Shea et al. 2011). We sutured GPS tags on three wild big brown bats (*Eptesicus fuscus*) during August and September of 2014 and inspected the condition of recaptured bats. Following our success with big brown bats, we proceeded to attach eight GPS tags (Pinpoint 8 with [*n* = 5] and without VHF transmitter [*n* = 3]) and six data loggers on wild hoary bats in Humboldt Redwoods State Park, California, in late September 2014.

Big brown bats in Fort Collins were captured in mist nets or harp traps while emerging from roosts in buildings. Two females were captured outside a colony located in a municipal park, and one male was captured at a bat house on the outside of a private residence. We captured hoary bats in mist nets along Bull Creek in Humboldt Redwoods State Park. The Bull Creek waterway was largely dry at time of capture, but hoary bats use it regularly as a flyway. Bats were captured in standard 2.6-m mist nets and in a triple-high configuration with three standard mist nets stacked on top of one another (Kunz and Racey 2009).

Nets and harp traps were constantly monitored to minimize the time bats were entrapped. Basic information recorded for each bat included relative age (juvenile vs. adult), sex, reproductive condition, body mass, and right forearm length. For both species, we attached tags to the individuals captured on a given night with the highest mass.

Bats chosen for tagging were gently restrained by hand, while the team veterinarian (KTC) assessed the bat’s suitability for anesthesia and transmitter placement (e.g., no open wounds, wing damage, or other injuries). Bats were held loosely in a cotton capture bag with only their head and dorsum exposed and were placed on a warming pad prior to and during anesthesia. Anesthesia was induced via face mask using 4.0–5.0% isoflurane in oxygen, delivered by precision vaporizer. Anesthesia was maintained via face mask using 1.5–4.0% isoflurane in oxygen. During anesthesia, the veterinarian monitored respiratory rate and quality, as well as response to stimuli, and adjusted isoflurane concentration as needed to maintain a safe and effective level of anesthetic depth.

**Figure 3:**
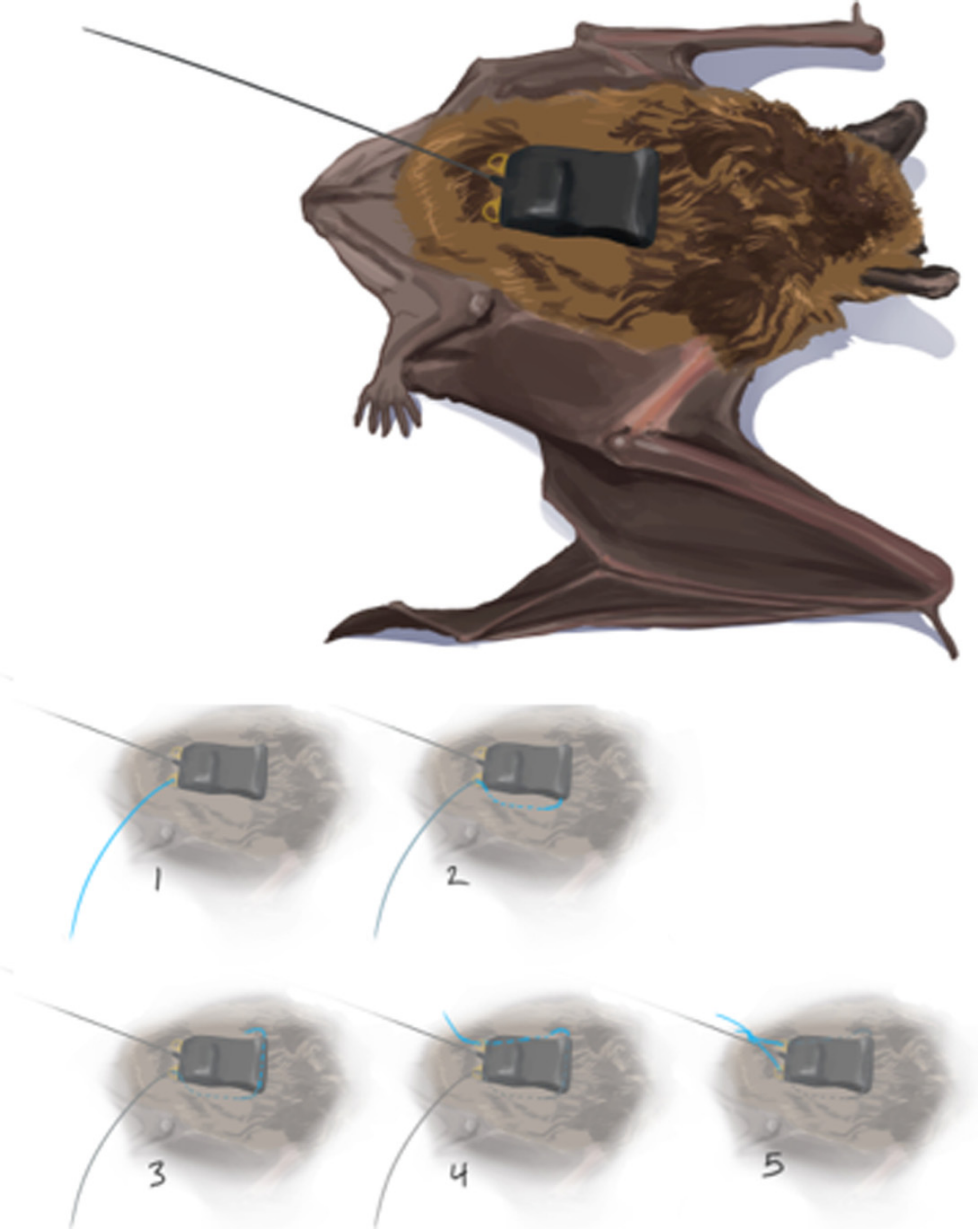
Diagram of suture placement to affix GPS tag to a big brown bat (*Eptesicus fuscus*). (1) Suture with needle is passed downwards through the loop at the back right of the tag and is put through the skin into the subcutaneous (SQ) space; (2) needle (& suture) is tunneled cranially in the SQ space and pulled out at the cranial aspect of the tag and passed through the “tube” in the tag; (3) needle is pulled out the left side of the “tube” and inserted into the SQ space and tunneled caudally through the SQ space to the caudal aspect of the tag; (4) needle is pulled out of the SQ space and upwards through the loop at the back left of the tag; and (5) ends of the suture are tied together using several knots thrown over the site where the antenna leaves the tag.

Once a bat was anesthetized, skin over the caudal dorsum was prepared using chlorhexidine scrub followed by an alcohol wipe. Optimum tag position (caudal to the scapulae and cranial to the pelvis) was determined visually and by palpation prior to suture placement. Each tag was affixed to the skin using 3-0 PDS II suture with a swaged (factory-attached), curved needle, in a horizontal mattress suture pattern (Fig.3). Due to the central location of the caudal attachment tube and caudally placed battery of the data logger tags, additional loops of suture were placed over the battery for better stabilization. Fit and position were checked prior to tightening the suture knots and discontinuing anesthesia.

After appropriate tag position and fit were confirmed, isoflurane was stopped, bats received 100% oxygen, and they were immediately placed back into their capture bag and kept warm until release. Bats always recovered and were ready for release after 15–30 min. When alert, each bat was held aloft in hand until it took flight on its own initiative.

In Colorado, autumn recapture attempts were made at 8–14 day intervals to capture bats carrying tags, examine them for injury, download location data, recharge batteries if necessary, and assess the fit of sutured devices. Upon recapture, big brown bats were anesthetized as previously described to allow close inspection of sutures and skin by the veterinarian and to assess the overall condition of the bat. After this brief physical examination, bats were allowed to recover from anesthesia and were gently restrained within a cotton holding bag, while location data were downloaded, the tag battery was recharged, and the GPS tag was reprogrammed. Tag programming and recharge were completed by connecting the GPS tag via a USB-powered programming and charging interface to a laptop computer, while the tag was still attached to the bat. Charging and programming of tags took 15–45 min depending on the number of previous fixes obtained. In addition to recaptures, the bat house containing the tagged bat was monitored continuously using an infrared video camera (model P1343, Axis Communications, Lund, Sweden) with supplemental near-infrared illumination (model IRLamp6; Wildlife Engineering, Tucson, AZ). This video monitoring allowed us to coarsely assess whether the GPS tag affected the bat’s ability to negotiate the small crevice openings of the bat house (approximately 1 cm).

In California, autumn recapture attempts were made six nights per week at a series of sites along an approximately 5-km reach of Bull Creek. Recapture was not attempted on nights with rain or >40% chance of rain. Recaptured bats were handled without anesthesia, and physical examinations and tag re-programming were conducted with bats gently restrained by hand. Upon recovery of tagged bats, GPS tags were downloaded, batteries recharged, and a new schedule of fixes assigned to the tag for redeployment. Bats carrying data loggers received a physical examination, tags were removed if detrimental effects (e.g., tag shifting, skin, or wing injury) were noted, and bats were released at point of capture.

Recapture attempts were made in Colorado and California during spring of 2015. Between March and May of 2015, we attempted to recapture big brown bats a total of three times at roosts where they were initially tagged. In California, we conducted 27 capture surveys between January 14 and May 14, 2015, 19 of which occurred between April 12 and May 14, 2015, to recapture tagged hoary bats at sites along Bull Creek.

## Results

We attached tags to three adult big brown bats in Colorado and 14 adult male hoary bats in California. After capture, anesthesia, and tag attachment, bats flew away normally (relative to bats simply captured and released); thus, the procedures did not appear to cause undue stress to bats, and no bats were injured during the process. Tags represented 4.9–6.4% of mass in big brown bats and 4.1–5.8% of mass in hoary bats (Table1).

**Table 1:** Summary data for GPS and data logger tags sutured to big brown bats [*Eptesicus fuscus* (EPFU)] and hoary bats [*Lasiurus cinereus* (LACI)] in Colorado and California, respectively, during late summer and autumn 2014

Tag ID and Type	Species	Date	Bat Mass (g)	Tag Mass (g)	Total Mass (g)	Tag % Body Mass
GPS 475	EPFU	8-Aug-14	22.5	1.1	23.6	4.9
GPS 476	EPFU	8-Aug-14	NA	1.1		NA
GPS 478	EPFU	21-Aug-14	18.8	1.1	19.9	6.4
GPS 477	LACI	26-Sep-14	20.7	1.1	21.8	5.3
GPS 479	LACI	22-Sep-14	21.5	1.1	22.6	5.1
GPS 481	LACI	26-Sep-14	23.1	1.1	24.2	4.8
VHF Tag 1	LACI	26-Sep-14	25.8	1.4	27.2	5.4
VHF Tag 2	LACI	27-Sep-14	24.5	1.4	25.9	5.7
VHF Tag 3	LACI	27-Sep-14	24.3	1.4	25.7	5.8
VHF Tag 4	LACI	27-Sep-14	25.1	1.4	26.5	5.6
VHF Tag 5	LACI	27-Sep-14	28.2	1.4	29.6	5.0
Data Logger Purple	LACI	26-Sep-14	23.2	1.1	24.3	4.7
Data Logger Red	LACI	26-Sep-14	22.2	1.1	23.2	4.9
Data Logger Blue	LACI	27-Sep-14	25.2	1.1	26.3	4.4
Data Logger Green	LACI	27-Sep-14	26.8	1.1	27.9	4.1
Data Logger Pink	LACI	26-Sep-14	22.5	1.1	23.6	4.9
Data Logger Yellow	LACI	27-Sep-14	23.0	1.1	24.1	4.8

During autumn, we recaptured five of the 11 bats equipped with GPS tags (two in Colorado, three in California) and three of the six bats to which data loggers were attached (Table2). We recaptured two big brown bats 11 and 12 days after tag attachment and did not observe tag movement or irritation on wings or skin of either bat. Three hoary bats were initially recaptured 2 days after device attachment, and single hoary bats were initially recaptured five, six, and 9 days following tag attachment (Table2). One hoary bat was recaptured a second time 9 days after initial tag attachment and another was recaptured a total of four times up to 26 days following initial device attachment. One tag (data logger) on a hoary bat was still sutured firmly to the skin, but had caused chafing to the skin under the tag, likely due to issues with the design of tag attachment points (Supporting Information). There was no bleeding or discharge from the chafed area, no sign of infection, and no indication of wing injury. That tag was removed, and the bat flew away normally when released. Data were downloaded successfully from all GPS tags, batteries were recharged while the tags were still on the bats, and each tag was reprogrammed to collect additional data. No sutures or knots had loosened. All GPS tags were left on the bats, and all recaptured bats flew away normally when released.

**Table 2:** Summary data for recaptures of *Eptesicus fuscus* (EPFU) and *Lasiurus cinereus* (LACI) in Colorado and California, respectively, to which GPS, GPS plus VHF, or data logger (DL) tags were sutured. Total mass includes bat and attached tag. “NA means “not applicable.”

Tag ID and type	Species	Date	Total mass (g)	Skin irritation?	Wing Injury?	Procedure	Days postattachment
GPS 475	EPFU	8-Aug-14	23.6	NA	NA	Initial attachment	NA
		20-Aug-14	Not weighed	No	No	Tag recharge and data download	12
GPS 478	EPFU	21-Aug-14	19.9	NA	NA	Initial attachment	NA
		1-Sep-14	19.3	No	No	Tag recharge and data download	11
		22-Mar-15	14.1	No	No	Tag Removal	227
		26-May-15	14.6	No	No	2 months postremoval	NA
VHF 5 (GPS + VHF)	LACI	27-Sep-14	29.7	NA	NA	Initial attachment	NA
		29-Sep-14	25.6	No	No	Recapture prior to fix attempt	2
		30-Apr-15	27.6	Yes	No	Tag Removal	213
GPS 481	LACI	26-Sep-14	24.2	NA	NA	Initial attachment	NA
		28-Sep-14	23.7	No	No	Recapture prior to fix attempt	2
		5-Oct-14	24.0	No	No	Tag recharge and data download	9
GPS 479	LACI	22-Sep-14	22.6	NA	NA	Initial attachment	NA
		28-Sep-14	24.0	No	No	Recapture prior to fix attempt	6
		5-Oct-14	21.2	No	No	Tag recharge and data download	9
		8-Oct-14	21.9	No	No	Recapture prior to fix attempt	12
		18-Oct-14	23.1	No	No	Tag recharge and data download	26
DL Pink	LACI	26-Sep-14	23.6	No	No	Initial attachment	NA
		28-Sep-14	23.2	No	No	Inspect and release	2
DL Purple	LACI	26-Sep-14	24.3	NA	NA	Initial attachment	NA
		1-Oct-14	22.1	No	No	Inspect and release	5
DL Blue	LACI	27-Sep-14	26.3	NA	NA	Initial attachment	NA
		6-Oct-14	24.0	Yes	No	Tag Removal	9
DL Yellow	LACI	27-Sep-14	24.1	NA	NA	Initial attachment	NA
		09-May-15	21.4	No	No	Tag Removal	224

During spring of 2015, we recaptured three bats after 213, 224, and 227 days, the former two from hoary bats in California and the latter from the male big brown bat using the bat house in Colorado. Tags were removed from all bats recaptured in spring 2015. Of the three tags recovered after more than 7 months of attachment, only the data logger attached to a hoary bat was still firmly attached upon recapture, and there was no evidence of fur loss, skin injury, infection, or wing injury from the sutures or the tag itself. A second tag (GPS + VHF) on a hoary bat had undergone significant rotation and was attached at only one corner by a thin piece of suture material; skin cranial to the tag was exposed but was not damaged. This tag likely would not have remained attached for many more days. That tag was removed and the bat flew away normally when released. A third tag (GPS) on the male big brown bat had worn away the fur beneath it and loose skin had bunched near the caudal end of the sutures, but upon removal, there was no sign of bleeding, bruising, or inflammation. When recaptured approximately 2 months later, the fur of this bat had not completely grown back where the tag had been and a 3-mm-diameter area of red, raised, flaked skin was observed near where the caudal sutures had been. However, the bat also exhibited skin flaking and areas of alopecia presumably associated with ectoparasite infection in areas distant from where the tag had been attached (e.g., on right side of cranium). It was unclear whether the irritation we observed was due to residual effects of the suturing or another skin condition.

Mean mass loss between tag attachment and initial recapture in hoary bats during autumn was 1.6 g (range: 0.3–2.1 g, *n *=* *6 bats). One bat (GPS 479) gained 1.4 g between attachment and initial recapture and exhibited both mass loss and gain in subsequent recaptures (Table2). The hoary bat (VHF 5) that carried a GPS+VHF tag over winter had an initial mass loss of 4 g (13.5%) 2 days after attachment but had recovered 2.0 g (6.8%) upon its recapture in spring. The hoary bat carrying the data logger over winter had a mass loss of 2.7 g (11.2%) relative to its initial capture. The single big brown bat for which we had data exhibited a mass loss of 0.5 g (3% of initial body mass) over an 11-night period of initial deployment, followed by an additional mass loss of 5.2 g over the winter that it carried the tag (Figure S1).

## Discussion

We developed a new method for long-term attachment of tracking devices to small bats (<30 g) that involves suturing devices directly to skin using absorbable sutures. With one minor exception, we did not observe adverse effects of tags to bat skin or wings. Bats were recaptured up to 7 months after tag attachment and all appeared healthy. Tracking devices performed reliably, justifying the use of this mildly invasive procedure to obtain important information on behavior and movements of bats that has previously not been possible. Our attachment method is especially promising because it ensures that the tags are secured close to the animal’s center of mass and away from highly dynamic body parts (e.g., wings, head, pelvis) that could result in interference with locomotion, skin irritation, and ultimately infection. Additionally, suturing the device to the skin at four points allows for a much closer fit to the body that should make it more aerodynamic. Suturing also has the potential to minimize snags on vegetation or roost structures, as well as avoid potential problems associated with rapid changes in angular momentum that could result from tags having fewer attachment points or being situated cranial to the bat’s center of mass. Although we were not able to evaluate tag attachment over a full year, this method appears to be a safe and effective alternative for long-term attachment of tags to bats under appropriate circumstances. Future technological developments are likely to include even lighter weight tags (<5% of mass) that will allow real-time global tracking and that will require longer attachment durations.

The suture material used in the present study was chosen because it maintains tensile strength considerably longer than most absorbable sutures and yet should be completely absorbed 182 – 238 days after placement in tissues (Ethicon product description). We were surprised to find that inspection of the sutures on recaptured bats, even 227 days after placement, suggested that the suture material would have lasted even longer. We speculate that suture life may be longer than manufacturer’s specifications because bats make frequent use of torpor, which depresses tissue temperature and other physiological processes that may help dissolve sutures. Assessing the duration of sutures under field conditions and hence predicting when a tag may be expected to fall off a bat will require additional study. More rapidly absorbing/dissolving suture materials are available, as are longer lasting (e.g., nonabsorbable synthetics) or permanent (stainless steel) suture materials, which could be used where the benefits of data collected outweigh the potential costs to bats if they cannot reliably be recaptured.

Weights of the GPS tags used in this study represented <6.4% of mass in big brown bats and <5.8% of mass in hoary bats, and weights of all data logger tags were <5% of body mass. The so-called 5% rule suggests that tag weight should be less than 5% of body mass in volant species to minimize the impacts of tags on movement and behavior (Aldridge and Brigham 1988; Amelon et al. 2009). Our study did not allow us to assess impacts of tag weight or configuration on flight and maneuverability; we tried to minimize any such impacts by tagging only the heaviest bats and by staying as close as possible to 5% of body weight. The fact that none of the recaptured bats exhibited unusual decreases in body mass and were recaptured up to 7 months after tag attachment indicates that our tag weights may not have caused unreasonable stress to our subject animals. Interestingly, the bat with the highest load (6.4%, EPFU 478; Table1) was recaptured after 7 months of wearing the tag, and its body mass during all recaptures (Table2) fell within the monthly average range of variation observed in adult males of that population (Figure S2). Future studies could obtain better data for assessing the 5% rule by observing the effects of sutured tags on larger samples of captive or wild bats.

The devices we attached showed great promise for improving understanding of seasonal movements and behaviors of individual bats. Such information has been recognized as vital to improving ecological understanding and informing conservation efforts (e.g., Holland and Wikelski 2009). The GPS tags we used successfully logged location data on bats moving at night in a variety of habitats and obtained daytime locations of roosting hoary bats. We measured single-night movements of 70 km in hoary bats and 33 km in big brown bats. Such distances generally exceed published measures based on VHF telemetry for these species (e.g., O’Shea et al. 2011; Bonaccorso et al. 2015) and at much lower cost and with lower human risk. The data logger tags we recovered showed close correspondence between measured activity patterns of hoary bats and light levels, thus demonstrating that nightly activity patterns of hoary bats can be revealed over long time periods using such devices. The fact that sunlight was reliably measured while the bat day-roosted also points to the promise of using lightweight geolocation devices to track migration of foliage-roosting bats (Fudickar et al. 2012). The success of the tags we deployed, especially as they are enhanced with future improvements in technology and reductions in tag mass, points to a rapid future increase in use of such devices to understand movements and behaviors of individual bats. As such, it is imperative that safe, reliable methods of tag attachment are used.

We evaluated potential impacts of tag attachment on bats solely on the basis of external inspection for injuries and change in body mass. Nevertheless, this is one of the few studies to report postattachment impacts to individual bats (Neubaum et al. 2005; Patriquin et al. 2010; O’Mara et al. 2014). Most bats to which we sutured tags had lost mass in the time since tag attachment. However, at our northern California site, seven of 17 hoary bats for which we confirmed recapture on the basis of PIT tags or wing punches during autumn of 2013 and 2014 also exhibited mass loss between initial marking and recapture (Supplemental Material). Mass fluctuations during late summer and autumn are common, particularly for male bats (Rughetti and Toffoli 2014). This point is underscored by mass gains and losses in individuals recaptured more than once in this study. The male big brown bat that was recaptured three times over 9 months after tagging showed no signs of seasonal mass loss beyond the range of variation typically seen in adult males of this population. Moreover, recaptures of hoary bats occurred away from roosts and at different times during the night; hence, mass at time of capture may have been influenced by amount of foraging or drinking prior to recapture.

Variable levels of skin mobility may lead to unwanted movement of the tag, leading to skin abrasion, as was seen with one of the data logger tags on a hoary bat in this study. Subjectively, the skin of hoary bats was much “looser” than that of big brown bats, and better fits were obtained on the latter. Positioning tags was more difficult with hoary bats due to skin movement during suture placement. Therefore, it is important to ensure the tag is properly situated and does not move as sutures and knot are tightened. In this study, a swaged, curved needle was used to pass suture through the skin and through attachment points on the tag. Using a straight needle may facilitate tag attachment in species with loose skin. Another potential issue with long-term suturing of tags to bat skin is molt. Both big brown bats and hoary bats undergo a single annual molt in late summer each year when the dorsal pelage is completely replaced (Cryan et al. 2004, 2012). In this study, we attached tags onto newly replaced fur and removed tags prior to summer molt, so were unable to assess whether fur growth or molt was affected by the sutured tag.

Despite our initial success with suturing tags to bats, this method should not be used without proper expertise and training. Anesthesia is required to minimize stress to the bat and properly position and suture tags, so veterinary expertise and portable anesthesia equipment are essential. Suturing experience is necessary to avoid injuring a bat through inappropriate suture placement (e.g., placing sutures into muscle or penetrating the body cavity) and to ensure that sutures are placed with optimal tension. We recommend that personnel with veterinary and surgical experience be enlisted to assist with these procedures. Once tags are attached, biologists should be able to recharge or remove sutured tags in the field without anesthesia or veterinary assistance. An important consideration with respect to the current generation of these technologies is that bats must be recaptured to retrieve data and tags. We selected field study sites where multiple previous years of field work indicated that recapture probabilities would be relatively high. We urge others considering similar work to carefully consider their ability to recapture animals prior to attaching tags to bats.

The goal of this study was to determine a safe, effective method of securing tracking devices to bats to measure broad-scale seasonal movements and behaviors over long time periods. Suture attachment shows great promise for maximizing retention periods while minimizing health impacts of tags on bats. Although we did not observe conspicuous negative impacts on bats, we note that more subtle negative impacts on survival probabilities and reproduction have been observed on birds that carried devices over multiple months (Scandolara et al. 2014). Such impacts will be more difficult to quantify in bats, but additional research that focuses on impacts of long-term tag attachment on bats is needed. Our method has the potential to meet these current and future needs and is an alternative to currently established methods (e.g., glue, collars) when a more aerodynamic, stable, and dependable tag attachment is required. This new attachment method should be particularly helpful in advancing our understanding of bat ecology as more sophisticated miniature tracking devices (e.g., satellite tags) become available.

## Data accessibility

Data available for download as Supporting Information.

## Supporting Information

**Data S1**. Materials.

**Video S1**. Representative video sequences showing an adult male big brown bat (*Eptesicus fuscus*) entering its usual roost in a bat house five nights before (first sequence), six nights after (second sequence), and 221 nights after (third sequence) having a GPS tag sutured to its back.

**Figure S1**. (A) Hoary bat (*Lasiurus cinereus*) with a data logger tag attached. Photo credit T. Weller. (B) Anesthetized big brown bat (*Eptesicus fuscus*) with a GPS tag attached. Photo credit P. Cryan.

**Figure S2**. Repeated body mass measurements of the adult male big brown bat (*Eptesicus fuscus*) tagged in this study (open triangles) compared to monthly average body mass (black circles with bars showing 95% confidence intervals) of adult males of the same species captured at nearby roosts during earlier studies (2001–2008) of this same population (O’Shea et al. 2011; T.J. O’Shea, pers. comm.).
