# Supplementary material for: Using sutures to attach miniature tracking tags to small bats for multimonth movement and behavioral studies
Source: Ecol Evol. 2015 Jul 4;5(14):2980–9. doi: 10.1002/ece3.1584 (PMC4542000; doi:10.1002/ece3.1584)
Supplement: Supplementary file 1 [file ece30005-2980-sd1.docx]

**Castle et al.**

**Supplemental Materials**

Additional Supporting Information may be found in the online version of this article.

***Considerations for suture attachment and the design of tracking tags.*** There was a distinct difference in the ease with which the various tags were attached, thus tag design improvements should be considered by manufacturers. The GPS-only tag design offered the easiest attachment and best fit due to the evenly-spaced attachment sites that consisted of two wire loops at the caudal end and a tag-width tube at the cranial end. The GPS-VHF tag design appeared adequate, however the tube at the front of the tag was narrower than the width of the tag and some rotation around the front end of the tag made it fit less flush to the skin surface. Among the three tags (one of each type) that were recovered more than seven months after attachment, the GPS-VHF tag was, by far, closest to detaching from the bat. Finally, the data-logger tag design, with two narrow attachment tubes, one located at the center and one on the cranial end of the tag, was the most difficult to attach. Because the battery was mounted external to the tag, much of the material and mass of the data-logger tag was located behind the middle attachment tube so the tag did not fit as close to the bat’s skin on initial attachment, particularly at the caudal end. Nevertheless the data logger tag recovered more than seven months after attachment was still snugly affixed to the bat.

***Anticipated benefits of suturing tags compared to collar attachment for long-term deployment.***

Longer-term attachment of tracking devices has been achieved using collars on bats (O’Mara, Wikelski & Dechmann 2014), however suturing tags to bats potentially offers a number of advantages over collars. Insectivorous bats sense their surroundings and acquire food by laryngeal echolocation, which requires complex and rapid production of sound through the throat and mouth. Placing a collar around a bat’s larynx for long periods has the potential to interfere with its navigation, foraging, or communication. Constricting the throat of a bat could also interfere with respiration and swallowing. Unlike birds, bats rely on rapid bi-directional breathing to meet the high metabolic demands of flight; constricting the airway with a collar could compromise a bat’s ability to maintain maximum physiological performance during flight. In addition, most bats roost in narrow crevices or dense foliage, leaving additional potential for a collared tracking device to snag on structures in the roost and entrap or injure the bat. Further potential issues with collar-attachment include: lateral force exerted on the bat's trajectory during quick turns and associated changes in angular momentum if the collar is rotating freely during flight; the transmitter flipping down to obstruct the bat's face or head while roosting upside-down or flying; and friction injuries caused by the continuous shifting of the collar. Attaching a device using a collar also places the forward mass of the tag against the base of the bat’s skull and directly over its shoulder blades. This placement has the potential to obstruct the highly mobile scapulae and shoulder girdle of bats during the upstroke of each wing beat and could possibly force the transmitter forward against the back of the bat's skull. Finally, sometimes dramatic seasonal changes in the body size and shape of bats associated with energy storage, reproduction, molt, or migration make it impossible to know whether a collar attached and adjusted at one point in time will later become too loose or tight if neck diameter changes. Although some of these problems could be addressed by gluing the tag to the bat's back in addition to the collar, the benefits of gluing would be temporary (several weeks) and the risks of collar-only attachment would return when adhesive failed.

***Consistent roost-entrance behavior and no unusual mass changes in tagged bats***

After initial tagging on 21 August 2014, the male big brown bat left the bat house and did not return until 27 August. From that day until it was recaptured on 1 September and again from 15 September until 23 September, it was observed on video entering and/or exiting the narrow crevices of the bat house on a near-nightly basis. After disappearing for the winter on 8 October 2014, it was again observed entering the bat house on the night of 16 March 2015. The speed and ease with which it entered the bat house were indistinguishable before and after tagging (Supplemental Video 1). In addition to video observations indicating no change in the way the bat entered and exited the bat house, repeated body mass measurements during recaptures fell within the range of variation previously observed in adult males of the same population (O’Shea et al. 2011; T.J. O’Shea, personal communication; Supplemental Figure 1).

During autumn 2013, we confirmed recapture of hoary bats based on the presence of a previous tissue biopsy punch hole in the left wing. We collected a tissue biopsy from the right wing and subsequent genotyping established the match between the captured individual and the recaptured individual. Five hoary bats were recaptured 1 day after initial capture and their mean mass change was +0.4 g (range -0.3 – 1.7 g). Three hoary bats were recaptured 7-8 days after initial capture and mass changes ranged from -2.0 – 2.2 g. Two hoary bats were recaptured 15 and 16 days after initial capture and their mass changes were -2.4 and 3.2g respectively. During autumn 2014 we inserted 0.1 g PIT tags into hoary bats to document recaptures. We recaptured 3 PIT-tagged bats 2 days after initial capture and found mass changes of -1.1, -0.4, and 1.5 g. Among PIT-tagged bats recaptured 13-15 days following initial tagging, we recorded mass changes of 0.7, -3.2, and -1.4 g respectively. In conclusion, mass changes observed in the hoary bats with suture-attached tags were generally within the range of those measured on bats that did not carry such payloads.

**SI Literature Cited**

O'Mara, M.T., Wikelski, M. & Dechmann, D.K.N. (2014) 50 years of bat tracking: device attachment and future directions. *Methods in Ecology and Evolution,* **5,** 311-319.

O'Shea, T.J., Neubaum, D.J., Neubaum, M.A., Cryan, P.M., Ellison, L.E., Stanley, T.R., Rupprecht, C.E., Pape, W.J. & Bowen, R.A. (2011) Bat ecology and public health surveillance for rabies in an urbanizing region of Colorado. *Urban Ecosystems,* **14,** 665-697.

Figure S1a. Hoary bat (*Lasiurus cinereus*) with a data logger tag attached. Photo credit T. Weller.

Figure S1b. Anesthetized big brown bat (*Eptesicus fuscus*) with a GPS tag attached. Photo credit P. Cryan.

**Figure S2.** Repeated body mass measurements of the adult male big brown bat (*Eptesicus fuscus*) tagged in this study (open triangles) compared to monthly average body mass (black circles with bars showing 95% confidence intervals) of adult males of the same species captured at nearby roosts during earlier studies (2001-2008) of this same population (O’Shea *et al.* 2011; T.J. O’Shea, personal communication). Note that for comparative purposes the dates on the X-axis (horizontal) are arranged chronologically by month, but not by year. We sutured a 1.1g GPS tag to the bat on 21 August 2014, recaptured and weighed the bat on 1 September 2014, and then it continued roosting in the bat house through 8 October 2014 when it departed for the year, presumably for its winter quarters. The bat was detected by video surveillance returning to the bat house on 16 March 2015 (Video S1, third sequence) and the tag was removed during the second recapture on 22 March 2015. The bat was captured one last time to assess its health on 26 May 2015, 65 days after the tag was removed. The mass of the tagged bat fell within the average ranges of monthly body weights for this population over the course of the 7 months that it carried the GPS device, and was about average two months after the tag was removed.

**Video S1.** Representative video sequences showing an adult male big brown bat (*Eptesicus fuscus*) entering its usual roost in a bat house five nights before (first sequence), six nights after (second sequence), and 221 nights after (third sequence) having a GPS tag sutured to its back. There was no noticeable change in the way the bat entered and exited the bat house after the tag was attached.
