# Supplementary figures and images for: Using sutures to attach miniature tracking tags to small bats for multimonth movement and behavioral studies
Source: Ecol Evol. 2015 Jul 4;5(14):2980–9. doi: 10.1002/ece3.1584 (PMC4542000; doi:10.1002/ece3.1584)

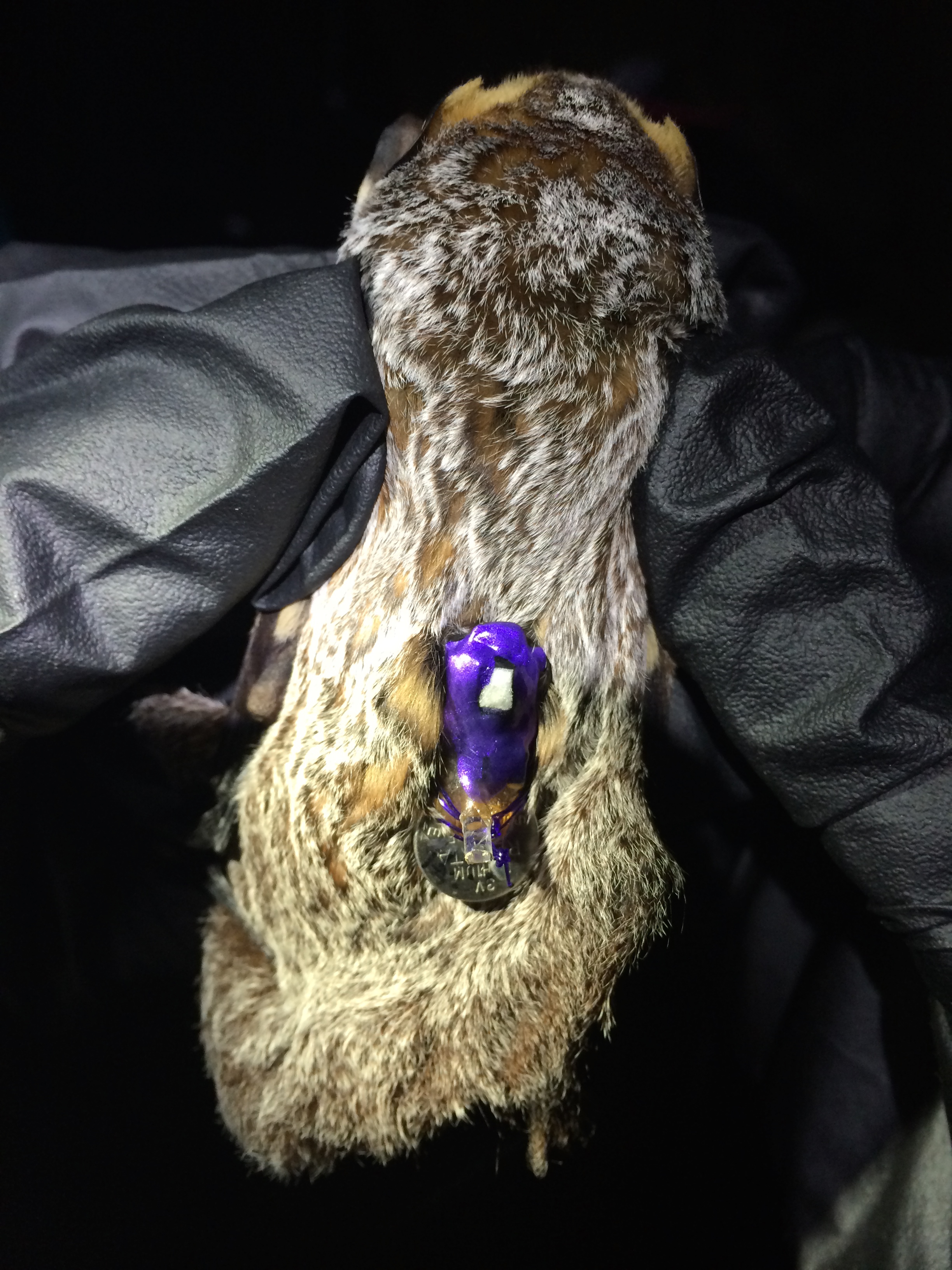

Supplement: Supplementary file 3 [file ece30005-2980-sd3.tiff]

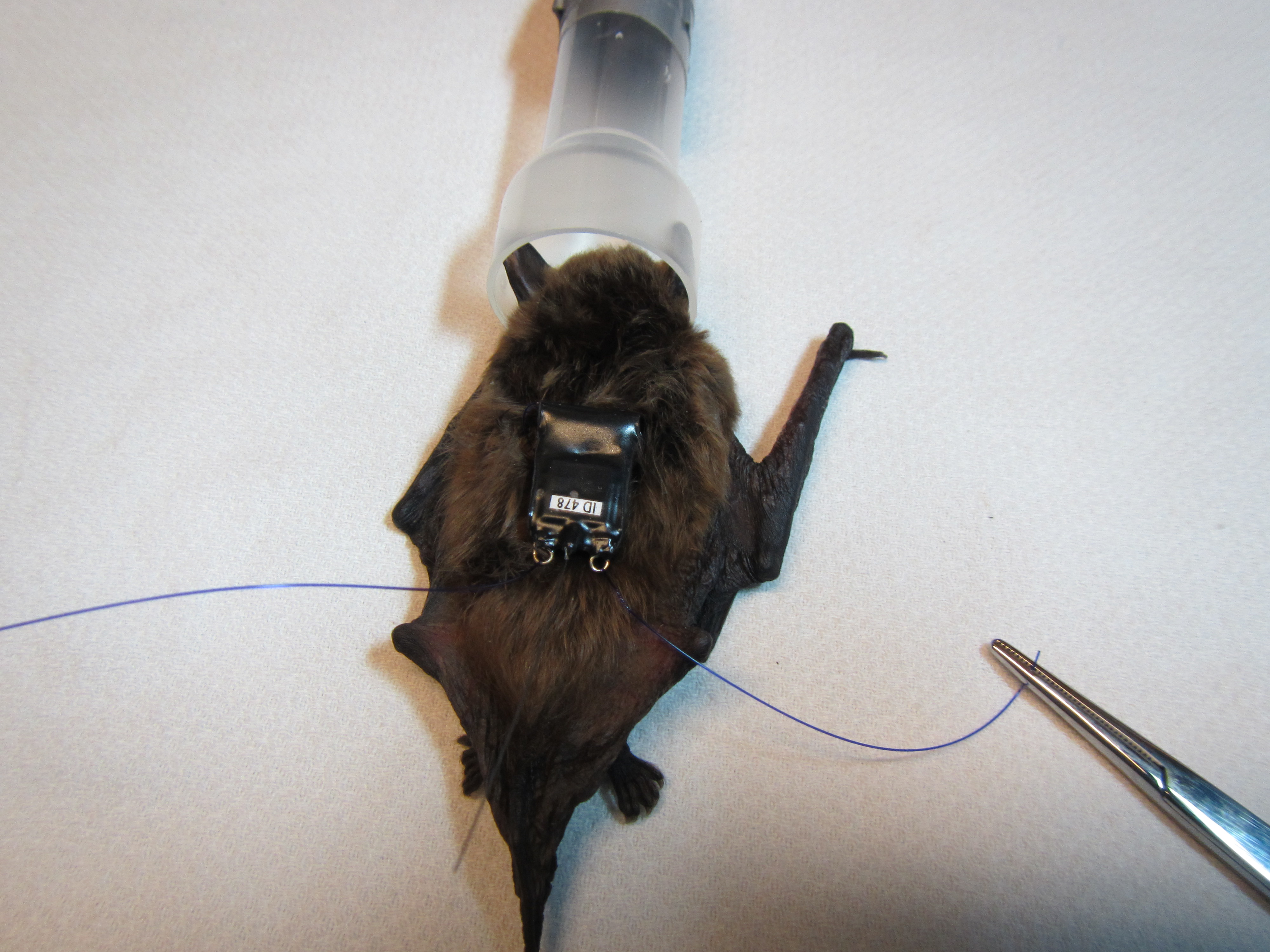

Supplement: Supplementary file 4 [file ece30005-2980-sd4.tiff]

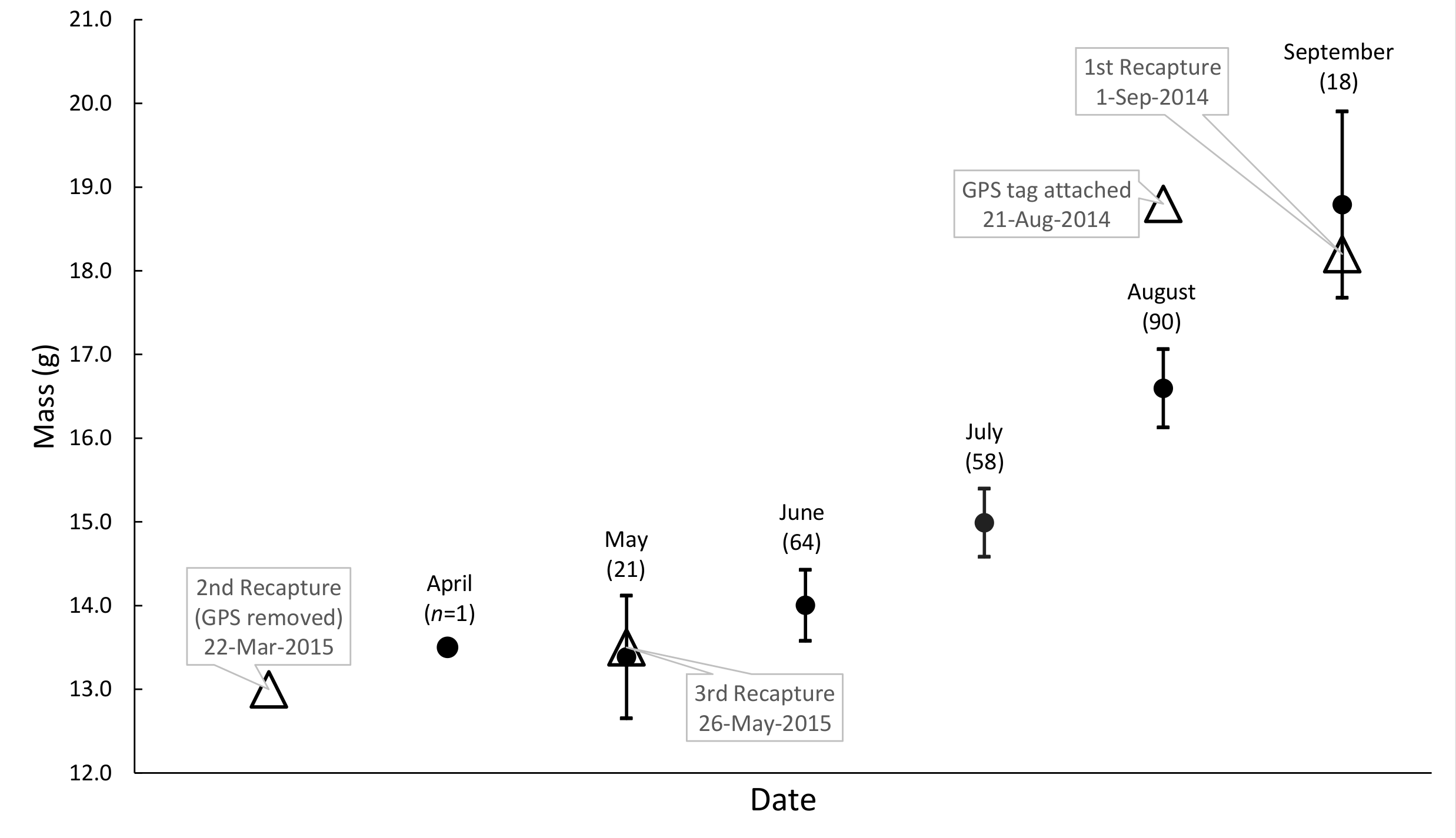

Supplement: Supplementary file 5 [file ece30005-2980-sd5.tiff]
